# Paradoxical gain‐of‐function mutant of the G‐protein‐coupled receptor PROKR2 promotes early puberty

**DOI:** 10.1111/jcmm.13146

**Published:** 2017-03-24

**Authors:** Maki Fukami, Erina Suzuki, Yoko Izumi, Tomohiro Torii, Satoshi Narumi, Maki Igarashi, Mami Miyado, Momori Katsumi, Yasuko Fujisawa, Kazuhiko Nakabayashi, Kenichiro Hata, Akihiro Umezawa, Yoichi Matsubara, Junji Yamauchi, Tsutomu Ogata

**Affiliations:** ^1^ Department of Molecular Endocrinology National Research Institute for Child Health and Development Tokyo Japan; ^2^ Department of Pharmacology National Research Institute for Child Health and Development Tokyo Japan; ^3^ Department of Pediatrics Hamamatsu University School of Medicine Hamamatsu Japan; ^4^ Department of Maternal‐Fetal Biology National Research Institute for Child Health and Development Tokyo Japan; ^5^ Department of Reproductive Biology Center for Regenerative Medicine National Institute for Child Health and Development Tokyo Japan; ^6^ National Research Institute for Child Health and Development Tokyo Japan

**Keywords:** GPCR, mutation, neuroendocrine

## Abstract

The human genome encodes ~750 G‐protein‐coupled receptors (GPCRs), including prokineticin receptor 2 (PROKR2) involved in the regulation of sexual maturation. Previously reported pathogenic gain‐of‐function mutations of GPCR genes invariably encoded aberrant receptors with excessive signal transduction activity. Although *in vitro* assays demonstrated that an artificially created inactive mutant of PROKR2 exerted paradoxical gain‐of‐function effects when co‐transfected with wild‐type proteins, such a phenomenon has not been observed *in vivo*. Here, we report a heterozygous frameshift mutation of *PROKR2* identified in a 3.5‐year‐old girl with central precocious puberty. The mutant mRNA escaped nonsense‐mediated decay and generated a GPCR lacking two transmembrane domains and the carboxyl‐terminal tail. The mutant protein had no *in vitro* signal transduction activity; however, cells co‐expressing the mutant and wild‐type PROKR2 exhibited markedly exaggerated ligand‐induced Ca^2+^ responses. The results indicate that certain inactive PROKR2 mutants can cause early puberty by enhancing the functional property of coexisting wild‐type proteins. Considering the structural similarity among GPCRs, this paradoxical gain‐of‐function mechanism may underlie various human disorders.

## Introduction

The human genome encodes ~750 GPCRs that possibly participate in various biological processes by transmitting extracellular signals to the interior of cells 1. To date, several loss‐of‐function mutations and a few gain‐of‐function mutations in GPCR genes have been identified in patients with congenital disorders, particularly endocrine and neuroendocrine diseases 1. Known pathogenic gain‐of‐function mutations in GPCR genes have invariably encoded hyperactive receptors with constitutive activity, low ligand specificity, excessive expression or defective desensitization 1, 2.

The hypothalamus–pituitary–gonadal axis comprises multiple GPCRs, including PROKR2, gonadotropin‐releasing hormone (GnRH) receptor and KISS1 receptor (KISS1R) 2. Loss‐of‐function mutations in genes for these GPCRs result in hypogonadotropic hypogonadism. In contrast, gain‐of‐function mutations in these genes have not been identified in human beings, except for the p.R386P mutation in *KISS1R* that was identified in a single patient with precocious puberty 3.

PROKR2 is a 384‐amino acid GPCR that dimerizes and regulates GnRH secretion in the hypothalamus 4, 5, 6, 7. Upon binding of the ligand prokineticin 2 (PROK2), PROKR2 couples with Gαq/11‐type G‐proteins, thereby mobilizing intracellular Ca^2+^. PROKR2 can also bind to Gαs‐ and Gαi/o‐type G‐proteins, although ligand‐induced signal responses of PROKR2 are more prominent in the Gαq/11‐mediated pathway than in the Gαs‐ and Gαi/o‐mediated pathways 8. *PROKR2* mutations account for a certain percentage of the aetiology of hypogonadotropic hypogonadism and Kallmann syndrome 4, 6, 9, 10. *In vitro* assays have confirmed functional impairment of several PROKR2 mutants 6. In addition, all known nonsense and frameshift mutations in *PROKR2* satisfied the conditions for nonsense‐mediated mRNA decay (NMD) 11. Recently, Sposini *et al*. 12 examined the functional property of artificially created PROKR2 variants. The authors found that a variant (TM1‐5) lacking the last two transmembrane domains and the carboxyl‐terminal tail exerts a unique effect on signal transduction; although the mutant retained no signal transduction activity, co‐transfection of this mutant markedly increased ligand‐induced signal responses of cells expressing wild‐type PROKR2. To date, however, such a paradoxical gain‐of‐function phenomenon of a GPCR has not been observed *in vivo*. In the present study, we identified a frameshift mutation in *PROKR2* in a patient with precocious puberty. This mutation encoded a GPCR structurally equivalent to the TM1‐5 variant.

## Materials and methods

### Patient

A 3.5‐year‐old girl was referred to our clinic because of early breast budding and accelerated statural growth. She was the sole child of non‐consanguineous Japanese parents. Physical examination revealed bilateral breast development of Tanner stage 2. Blood analyses demonstrated markedly elevated oestradiol and hyper‐responses of gonadotropins to GnRH (Table S1). Bone age was advanced (4.7 years of age). Brain magnetic resonance imaging revealed no abnormalities. The girl was diagnosed with idiopathic central precocious puberty and underwent GnRH analogue treatment from 3.7 to 11.4 years of age. During this treatment, her sexual development was suppressed. She had menarche at 13.0 years of age and regular menses thereafter. Her parents were clinically normal. The mother had menarche at 12 years of age (mean menarcheal age in Japanese women is 12.25 years). Allegedly, no maternal relatives of the patient had a history of precocious puberty.

### Molecular analyses

This study was approved by the Institutional Review Board Committee at the National Center for Child Health and Development and was performed after obtaining written informed consent. This study was carried out according to the World Medical Association Declaration of Helsinki. Detailed methods are shown in the Supporting Information of Data S1. We performed molecular analysis of the patient's genomic DNA. Twenty‐nine genes involved in the regulation of GnRH secretion were sequenced. Copy‐number abnormalities in the genome were examined by array‐based comparative genomic hybridization. We also analysed parental DNA and the patient's mRNA.

To clarify the functional consequences of a *PROKR2* mutation identified in the patient, we performed Ca^2+^ mobilization analyses. Chinese hamster ovary‐K1 cells were transiently transfected with the expression vectors for the wild‐type and/or mutant PROKR2. Ca^2+^ responses were measured before and after ligand stimulation.

## Results

**Figure 1:**
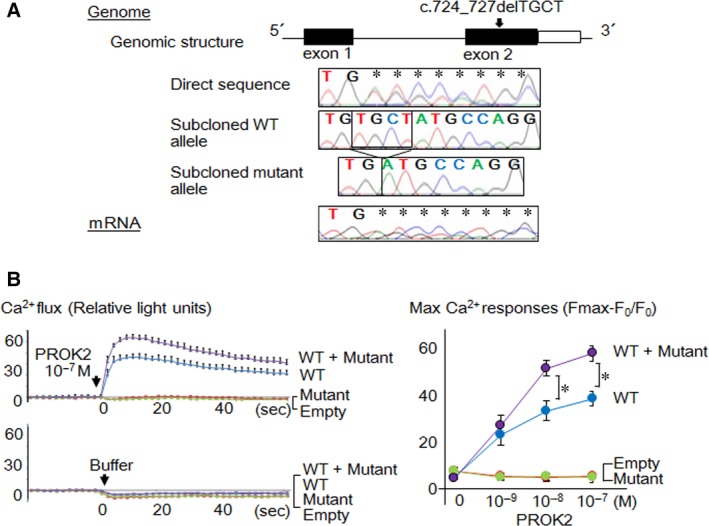
Identification and characterization of the *PROKR2* mutation. (**A**) Results of the molecular analyses. Black and white boxes in the genomic structure depict coding and non‐coding exons, respectively. The girl carried a heterozygous c.724_727delTGCT mutation in *PROKR2* exon 2. RT‐PCR revealed the presence of both wild‐type (WT) and mutant mRNA. (**B**) Representative results of Ca^2+^ mobilization assays. The left panel shows Ca^2+^ flux curves of cells transfected with WT alone (blue lines), mutant alone (green lines), both wild‐type and mutant (WT + Mutant, purple lines), and an empty vector (red lines) (the mean ± 1.0 standard deviation). The cells were treated with 10^−7^ M PROK2 or buffer (Hank's balanced salt solution plus HEPES buffer). The right panel shows max Ca^2+^ responses to different doses of PROK2 (0, 10^−9^, 10^−8^ and 10^−7^ M) calculated from the ratio between the change in fluorescence signal intensity (Fmax‐F_0_) and the baseline intensity (F_0_). Asterisks indicate significant differences between WT alone and WT + Mutant (*P* ≤ 0.05).

A heterozygous 4‐bp deletion in *PROKR2* (c.724_727delTGCT, p.C242fsX305) was identified in the girl and her mother (Fig. 1A). The girl had no additional mutations or copy‐number alterations. The *PROKR2* mutation was hitherto unreported and was absent from 121,412 alleles in the exome database. RT‐PCR products from the girl contained almost equal amounts of wild‐type and mutant *PROKR2* alleles, indicating that the mutant mRNA escaped NMD (Fig. 1A).

Ca^2+^ mobilization assays revealed paradoxical gain‐of‐function effects of the mutant PROKR2 (Fig. 1B). Basal signal activity was similar among cells transfected with empty and PROKR2 expression vectors. Cells transfected with wild‐type PROKR2 showed ligand‐induced Ca^2+^ flux, while cells transfected with the mutant did not respond to ligand stimulation. When treated with >10^−8^ M PROK2, cells co‐transfected with both proteins showed significantly greater Ca^2+^ responses than cells transfected with the wild‐type alone. The Ca^2+^ flux curves of the wild‐type‐expressing cells and those of the co‐expressing cells showed parallel changes after ligand administration.

## Discussion

We identified a heterozygous p.C242fsX305 mutation in *PROKR2* in a girl with precocious puberty. Mutant mRNA escaped NMD and appeared to encode a truncated PROKR2 lacking two transmembrane domains and the carboxyl‐terminal tail. Although the mutant protein retained no *in vitro* signal transduction activity, cells co‐expressing the wild‐type and the mutant showed greater ligand‐induced Ca^2+^ flux than cells expressing the wild‐type alone. Thus, p.C242fsX305 likely enhances the activity of the coexisting wild‐type receptor. Additionally, p.C242fsX305 may suppress desensitization of the receptor dimers, because the mutant lacked the carboxyl‐terminal tail that mediates receptor internalization 13. However, as Ca^2+^ flux curves of the co‐transfected cells paralleled that of the wild‐type‐transfected cells, enhanced signal transduction rather than delayed desensitization appears to be the major effect of p.C242fsX305. These results are consistent with those of a previous study by Sposini *et al*. 12. Our p.C242fsX305 mutant may modify the spatial arrangement of PROKR2 protomers to form hyperactive heterodimers, as in the case of the TM1‐5 variant 12.

The phenotype of our patient is compatible with the excessive PROKR2 function in the hypothalamus 6. As this patient exhibited regular menstrual cycles after 13.0 years of age, gain‐of‐function of PROKR2 unlikely affects feedback regulation of the hypothalamus–pituitary–gonadal axis. Notably, none of the patient's family members, including the mother with the same mutation as the patient, manifested apparent precocious puberty. These results can be explained by assuming that the clinical consequences of this mutation are determined by the balance between hetero and homodimers in the cells; a relatively small proportion of PROKR2 proteins may have formed heterodimers in the hypothalamic cells of the mother. Alternatively, the phenotypic variation in this family may reflect the incomplete penetrance or variable expressivity of *PROKR2* abnormalities, because previously reported *PROKR2* mutations were associated with a wide phenotypic spectrum 4, 6, 9, 10.

This study has two limitations. First, we did not examine whether p.C242fsX305 actually dimerizes to the wild‐type PROKR2 *in vivo*. Thus, although Sposini *et al*. 12 have shown that the TM1‐5 variant dimerizes with the wild‐type protein, the physical interaction between p.C242fsX305 and wild‐type PROKR2 needs to be confirmed by future studies. Second, subcellular localization of the wild‐type and mutant PROKR2 was not examined this study. Thus, we cannot exclude the possibility that p.C242fsX305 exerts a high signal transduction activity by affecting the internalization of the heterodimerized wild‐type protein.

Collectively, our results indicate that some inactive PROKR2 mutants can cause precocious puberty by enhancing the functional property of coexisting wild‐type proteins. This notion challenges the current understanding that all pathogenic gain‐of‐function mutations in GPCR genes encode hyperactive receptors 1. Considering the structural similarity among the ~750 known GPCRs 1, this paradoxical gain‐of‐function mechanism may underlie various human disorders.

## Conflict of interest

The authors confirm that there are no conflicts of interest.

## Author contributions

MF, JY and TO designed the study; MF, ES, YI, TT, SN, MI, MM, MK, KN, KH, AU, YM and JY performed molecular analyses; MF, ES and SN analysed the data; YF and TO collected clinical data; and MF wrote the manuscript.

## Supporting information


**Table S1** Hormonal findings of the girl

**Data S1** Supporting Methods
