# Supplementary material for: Paradoxical gain‐of‐function mutant of the G‐protein‐coupled receptor PROKR2 promotes early puberty
Source: J Cell Mol Med. 2017 Mar 24;21(10):2623–6. doi: 10.1111/jcmm.13146 (PMC5618689; doi:10.1111/jcmm.13146)
Supplement: Supplementary file 1 — Table S1 Hormonal findings of the girl [file JCMM-21-2623-s001.docx]

**Table S1**

| Table S1. Hormonal findings of the girl. | | | |  |  |
| --- | --- | --- | --- | --- | --- |
|  | Estradiol  (pg/mL) | Luteinizing hormone  (mIU/mL) | | Follicle stimulating hormone (mIU/mL) | |
|  |  | Basal | Stimulated^a^ | Basal | Stimulated^a^ |
| At 3.5 years of age | | | | | |
|  | **60.7** (< 15.0) | **0.52** (0.01-0.09) | **7.20** (1.93-4.73) | **7.70** (0.54-2.47) | **51.0** (10.7-38.1) |
| At 13.0 years of age | | | | | |
|  | 45.0 (15.0-60.0) | 10.40 (0.82-11.74) | no data | 5.60 (2.61-6.64) | no data |
| Reference values of age-matched Japanese females are shown in parenthesis. | | | | |  |
| Hormone values above the reference range are boldfaced. | | | |  |  |
| ^a^ Gonadotropin releasing hormone stimulation test. | | | |  |  |
