# Supplementary material for: Paradoxical gain‐of‐function mutant of the G‐protein‐coupled receptor PROKR2 promotes early puberty
Source: J Cell Mol Med. 2017 Mar 24;21(10):2623–6. doi: 10.1111/jcmm.13146 (PMC5618689; doi:10.1111/jcmm.13146)
Supplement: Supplementary file 2 — Data S1 Supporting Methods [file JCMM-21-2623-s002.docx]

**Supporting Methods**

***Molecular analyses***

This study was approved by the Institutional Review Board Committee at the National Center for Child Health and Development, and was performed after obtaining written informed consent. Detailed methods are shown in the Supplementary Information. A genomic DNA sample of the girl was obtained from peripheral leukocytes. We searched for protein-altering substitutions and splice-site mutations in 28 genes that have been implicated in the gonadotropin regulation, namely, *CHD7*, *FGF8*, *FGFR1*, *FSHB*, *GNRH1*, *GNRHR*, *HESX1*, *KAL1*, *KISS1*, *KISS1R*, *LEP*, *LEPR*, *LHB*, *LHX3*, *LHX4*, *NELF*, *NR0B1*, *OTX2*, *POU1F1*, *PROK2*, *PROKR2*, *PROP1*, *SEMA3A*, *SOX2*, *SOX3*, *TAC3*, *TACR3*, and *WDR11* [1]. A DNA library was created using the Haloplex system (Agilent Technologies, Palo Alto, CA, USA) and sequenced on a MiSeq next generation sequencer (Illumina, San Diego, CA, USA). Nucleotide alterations were called by the Avadis NGS 1.3.1 (DNA Chip Research, Yokohama, Japan) or SAMtools 0.1.17 software (http://samtools.sourseforge.net/). Putative pathogenic mutations indicated by next-generation sequencing were confirmed by the Sanger method. In addition, mutations in *MKRN3*, a newly identified causative gene for central precocious puberty [2], were analyzed by Sanger sequencing. Primer sequences are available upon request.

To confirm a heterozygous mutation in *PROKR2*, we subcloned PCR products carrying the mutation into the TOPO TA cloning vector (Life Technologies, Carlsbad, CA, USA) and the mutant and wildtype alleles were sequenced separately. Genomic DNA samples obtained from the patient’s parents were examined for the presence or absence of the *PROKR2* mutation. We referred to databases (NCBI, http://www.ncbi.nlm.nih.gov/; and Exome Aggregation Consortium Browser, http://exac.broadinstitute.org/) to examine whether this mutation has been identified in general populations.

To examine whether the *PROKR2* mutation induces nonsense-mediated mRNA decay, we analyzed the patient’s mRNA sample. Total RNA was obtained from peripheral leukocytes using the RNeasy Plus Mini Kit (Qiagen, Valencia, CA, USA). RT-PCR was performed using primers that hybridize to exons 1 and 2 of *PROKR2*. RT-PCR products were subjected to direct sequencing.

We also analyzed copy-number abnormalities in the genome. Array-based comparative genomic hybridization (CGH) was performed using a catalog human CGH array (4 × 180 k format, catalog number G4449A; Agilent Technologies), according to the manufacturer’s instructions. We referred to the Database of Genomic Variants (http://projects.tcag.ca/variation/) to exclude benign copy-number variants.

***Plasmids***

Expression vectors for wildtype PROKR2 were constructed by inserting cDNA fragments of *PROKR2* into the pTagGFP2-N vector (Evrogen, Moscow, Russia). The cDNA fragments were amplified by PCR using a plasmid containing wildtype *PROKR2* cDNA (catalog number FHC01750; Kazusa DNA Research Institute, Kisarazu, Japan) as the template. An expression vector for the mutant PROKR2 was generated by mutagenesis using the PrimeStar Mutagenesis Kit (Takara Bio, Otsu, Japan).

***Ca^2+^ mobilization assays***

Chinese hamster ovary-K1 cells (CHO-K1; ATCC, Manassas, VA, USA) were cultured in 6-well plates (5.0 × 10^5^ cells/plate). The cells were transiently transfected with expression vectors for wildtype PROKR2 and/or those for the mutant protein (total 5.0 μg/plate). As a negative control, the same amount of the empty expression vector was transfected. At 24 hours after transfection, the cells were collected and re-seeded in 96-well black-wall clear-bottom plates (4.0 × 10^4^ cells/well), and incubated for a further 24 hours. Then, Ca^2+^ assays were performed using the FLIPR Calcium 6 Assay Kit and FlexStation (Molecular Devices; Sunnyvale, CA, USA), according to the manufacturer’s instructions. Various doses of PROK2 or buffer (Hank’s Balanced Salt Solution plus HEPES buffer) was automatically added to each well. Ca^2+^ flux was calculated from the average of the values from four wells. All experiments were repeated three times. Peak relative light units were calculated from the average of four wells. Statistical significance was determined by the *t*-test.

**Reference**

1. **Izumi Y, Suzuki E, Kanzaki S, *et al.*** Genome-wide copy number analysis and systematic mutation screening in 58 patients with hypogonadotropic hypogonadism. *Fertil Steril.* 2014; 102: 1130–6.
2. **Abreu AP, Dauber A, Macedo DB, *et al.*** Central precocious puberty caused by mutations in the imprinted gene MKRN3. *N Engl J Med.* 2013; 368: 2467–75.
